# Is potentially inappropriate prescribing in UK middle-aged adults associated with healthcare utilisation and mortality? A prospective cohort study of a 1.2 million patient cohort conducted in the clinical practice research datalink

**DOI:** 10.1136/bmjopen-2025-115606

**Published:** 2026-07-27

**Authors:** Michael Naughton, Mariam Molokhia, Patrick Redmond, Ian J Douglas, Rohini Mathur

**Affiliations:** 1 The Clinical Effectiveness Group, The Wolfson Institute of Population Health, Queen Mary University of London, London, England, UK; 2 Faculty of Epidemiology and Population Health, London School of Hygiene & Tropical Medicine, London, England, UK; 3 School of Population Health & Life Course Sciences, King’s College London, London, England, UK; 4 Department of General Practice, Royal College of Surgeons in Ireland, Dublin, Leinster, Ireland; 5 Clinical Effectiveness Group, The Wolfson Institute of Population Health, Queen Mary University of London, London, England, UK

**Keywords:** Primary Care, CLINICAL PHARMACOLOGY, EPIDEMIOLOGIC STUDIES, Mortality, Drug Therapy, Health Services

## Abstract

**Abstract:**

**Objectives:**

To estimate the prevalence of potentially inappropriate prescribing (PIP) in UK middle-aged adults, identify patient-level factors associated with PIP and its associations with subsequent primary care utilisation and mortality.

**Design:**

A prospective observational cohort study.

**Setting:**

English routinely collected primary care electronic health record data from the Clinical Practice Research Datalink Aurum. The follow-up period was from 1 January 2018 to 7 February 2023.

**Participants:**

Randomly selected individuals (n=1 200 000) who were aged 45–64 years on 1 January 2018.

**Exposure:**

Exposure was defined as at least one episode of PIP in the year 2018.

**Primary and secondary outcomes:**

The primary outcome was the rate of GP consultations in the year following exposure (2019). The secondary outcome was mortality between 2019 and 2023.

**Results:**

13.9% of participants experienced PIP in 2018. Factors significantly associated with PIP included polypharmacy (≥10 medications: OR 6.93, 95% CI 6.61 to 7.27), multimorbidity (OR 2.47, 95% CI 2.45 to 2.50), female sex (OR 1.14, 95% CI 1.13 to 1.16), older age (OR 1.01 95% CI 1.01 to 1.02) and higher primary care consultation frequency (OR 1.04 95% CI 1.04 to 1.04). PIP was associated with increased primary care consultations the following year (IRR 1.03, 95% CI 1.02 to 1.03) but not mortality (HR 0.99, 95% CI 0.93 to 1.04).

**Conclusion:**

One in seven middle‐aged adults were exposed to PIP and PIP resulted in higher subsequent primary care consultations; medicine optimisation strategies for PIP warrant evaluation in middle aged adults.

**STRENGTHS AND LIMITATIONS OF THIS STUDY**

- This study was carried out in the Clinical Practice Research Datalink, a large national primary care database with quality assured data on a random middle-aged adult sample representative of national UK population. This means the findings are likely to be generalisable to the UK population.
- Covariates and confounders in models were prespecified based on literature and mapped using a direct acyclic graph, minimising overfitting and investigator bias.
- Multilevel regression models were used, so the results are adjusted for area level and practice–practice prescribing variability.
- There is likely to be residual confounding due to the observational nature of the study and the fact that patient and area level socioeconomic deprivation was not adjusted for in regression models.
- As linked data were unavailable, we were unable to look at secondary care healthcare utilisation and the definition of mortality relied on Primary Care coding rather than Office for National Statistics (ONS) data.

## Introduction

 The scale and complexity of prescribing in UK primary care continue to rise. Over one billion items (medicines and devices) are now dispensed annually in England, increasing by 2%–3% yearly, driven by an ageing population and chronic disease management.^1^ Recent decades have seen pronounced shifts in prescribing; antidepressant prescriptions have more than tripled between 1998 and 2018, reaching 1266 items per 1000 people per year,^2^ prescribing of direct oral anticoagulants have similarly increased.^3^ Polypharmacy—commonly defined as the use of five or more regular medications—affects 27% of all adults and over half of those aged 55–64.^4^ While regimens may be justified in managing long-term conditions, the risk of drug–drug interactions and cumulative adverse effects increase substantially with polypharmacy. Concurrently, many patients experience treatment burden—with complex regimens, side effects and medication reviews. National audit data suggest that at least 10% of prescriptions confer no clear clinical benefit, highlighting a need for proactive medicines optimisation.^5 6^

### Defining potentially inappropriate prescribing

Potentially inappropriate prescribing (PIP) refers to the use of medicines where potential harms outweigh likely benefits, safer alternatives exist or necessary treatments are omitted.^7^ PIP can be identified using explicit tools such as the Beers^8^ and STOPP/START criteria^9^—developed for older adults—or the Prescribing Optimally in Middle-aged People’s Treatments (PROMPT) criteria, specifically designed for individuals aged 45–64.^10^

Implicit tools such as the Medication Appropriateness Index exist, though are more dependent on clinical judgement.^11^ In older populations, PIP is consistently associated with increased hospitalisations, emergency department use, falls and avoidable healthcare costs.^12–15^

### Why focus on adults aged 45–64?

Prescribing safety initiatives typically focus on older populations (≥65 years), yet a significant burden of multimorbidity and polypharmacy now exists in mid-life (45–64 years).^4 16 17^ Multimorbidity and polypharmacy in mid-life intersect with sociodemographic characteristics such as deprivation; one-third of adults in the most socioeconomically deprived areas of the UK have multiple chronic conditions by their early 50s.^18–20^ A recent UK study found 14% of adults aged 45–64 are prescribed five or more regular medications, and 4% receive 10 or more.^20^

International studies report PIP prevalence estimates in this age group ranging from 5% to 57%, with a pooled average of 38%.^21–23^ However, UK-specific data remain limited, and previous studies have been underpowered or geographically restricted. A small Irish study suggests that PIP in middle age is associated with adverse drug reactions, but evidence linking PIP to service utilisation or mortality remains inconclusive.^24 25^

### Unanswered questions and equity considerations

Three key gaps remain. First, robust UK estimates of PIP prevalence in middle-aged adults are lacking. Second, while PIP in older adults is associated with female sex, multimorbidity, deprivation and polypharmacy,^13 26–29^ it is unclear if these associations hold in mid-life, and no UK study has examined the association between ethnicity and PIP in adults aged 45–64. This is notable given emerging evidence of disparities in prescribing quality and medication safety among ethnic minority groups.^30 31^ Finally, the potential impact of PIP on primary care workload and patient outcomes is unknown in this age group.

These gaps limit the evidence base for extending medicines optimisation programmes to those in mid-life, who may also be at risk of avoidable harm.

### Aims and objectives

This study aimed to address these gaps by utilising routinely collected data from Clinical Practice Research Datalink (CPRD) Aurum. We sought to:

1. Estimate the prevalence of PIP among UK middle-aged adults using PROMPT criteria.
2. Identify patient-level factors, including age, sex, ethnicity, multimorbidity, polypharmacy and healthcare utilisation patterns, associated with PIP.
3. Examine the associations of PIP with primary care consultation frequency in the subsequent 12 months and all-cause mortality till end of follow-up (7 February 2023).

## Methods

### Study design and reporting

We conducted our cohort study according to observational routinely collected health data statement for pharmacoepidemiology (RECORD-PE) guidelines.^32^ The observation period was from 1 January 2018 to 07 February 2023, with exposure to PIP defined 1 January 2018–31 December 2018.

### Data source

Data were obtained from the CPRD Aurum, a database containing pseudonymised primary care records from English general practices using EMIS software. CPRD Aurum is broadly representative of the English population regarding age, sex, ethnicity, deprivation, and geographical distribution.^33 34^

### Study population

We identified a random sample of 1 200 000 adults aged 45–64 years as of 1 January 2018. Participants were required to have continuous registration with the same CPRD Aurum practice January 2018–December 2019. Individuals who transferred practices or died before the end of the exposure definition period (31 December 2018) were excluded prior to sampling. The population size was selected to have a>80% power to detect a 10% difference in mortality (p<0.05), as the most important but also rarest outcome.

### Exposure definition

Exposure to PIP was defined as being exposed to at least one PROMPT criteria during 2018. These were constructed using issued prescription data and diagnosis codes extracted from CPRD. The PROMPT list comprises 22 explicit prescribing scenarios considered PIP for middle-aged adults (construction method for criteria detailed in online supplemental table S1.^10^ Exposure status was treated primarily as binary (exposed/unexposed), a count of criteria exposure was used for sensitivity analyses.

### Outcomes

The primary outcome was rate of primary care consultations recorded during the year following exposure (1 January–31 December 2019). The secondary outcome was all-cause mortality, identified through recorded death dates in CPRD Aurum, from 1 January 2019 to either patient deregistration or end of follow-up (7 February 2023).

### Variables and definitions

Age, sex and self-reported ethnicity (white, South Asian, black, Mixed, Other and not stated) were defined at baseline (1 January 2018). Polypharmacy was defined as the number of distinct British National Formulary (BNF) subchapters prescribed in the first months of the observation period (1 January 2018–31 March 2018), missing BNF Chapter was reversed engineered using product code and drug record identifier code. Multimorbidity was assessed using the Cambridge Multimorbidity Score (general outcome weighted score), incorporating 20 long-term conditions,^35^ individuals were counted as having a diagnosis if they have ever been diagnosed using one of the appropriate codes prior to enrolment in the study. Primary care consultation frequency during 2018 included all consultation modalities. When we looked at factors associated with PIP, these were treated as potential explanatory variables, for PIP association with outcomes, they were potential confounders.

### Statistical analysis

Statistical analyses were performed using Stata (V.18).^36^ Descriptive statistics summarised baseline characteristics, reporting categorical data as counts and percentages, and continuous variables as means (SD) or medians (IQR), based on distribution. Multiple imputation by chained equations addressed missing ethnicity data,^37^ using univariate method with multinomial logistic regression to impute ethnicity category and 10 iterations carried out. 13 intersex individuals were excluded from analysis due to small numbers, other variables had no missing data. Models were tested for interactions and multiple collinearity. Multilevel models were used with patients grouped by practice using a random intercept model, to allow for practice level different clinician prescribing practices.

### Prevalence of PIP

Annual PIP prevalence was calculated by the number of individuals experiencing at least one PIP indicator during the period 1 January 2018 till 31 December 2018 divided by the total of patients within the cohort. Prevalence of each of the individual indicators was also calculated.

### Factors associated with PIP

A two-level multilevel logistic regression model, with patients nested within practices, was used to estimate associations between patient-level factors (sex, age, ethnicity, multimorbidity, polypharmacy, annual consultation frequency) and PIP exposure, accounting for clustering by practice. Fully adjusted ORs with 95% CIs were reported.

### Associations between PIP and consultation frequency

A negative binomial regression model assessed the association between PIP exposure in 2018 and primary care consultation counts in 2019. This was chosen as consultation rate data were over-dispersed, violating a Poisson regression model assumption. A fully adjusted model included practice-level clustering, age, sex, ethnicity, polypharmacy, multimorbidity and consultations the previous year (2018).

### Associations between PIP and mortality

Cox proportional hazards regression evaluated the association between PIP exposure and all-cause mortality. Follow-up commenced on 1 January 2019, ending at the earliest of death, practice deregistration or the end of follow-up (7 February 2023). Analyses were adjusted for practice, age, sex, ethnicity, polypharmacy, multimorbidity and consultations in the year prior to follow-up.

### Sensitivity analyses

The following sensitivity analyses were conducted to test robustness of the primary analyses:

1. Modelling the count of PROMPT criteria using multilevel Poisson regression (online supplemental table S3).
2. Conducting a complete-case analysis (online supplemental table S4).
3. Repeating analyses after excluding individuals with high consultation frequency (>20 consultations) in 2018 (online supplemental table S5).
4. Mortality restricted to follow-up to 31 December 2019, to minimise potential confounding related to the COVID-19 pandemic (online supplemental table S6).

## Results

In our sample of 1.2 million adults aged 45–64, the population had more men than women (51.1%). A multilevel regression model was used with missing data imputed using chained equations, so the entire population sample was used for analysis. The mean age was 53.9 years (SD 5.58). 64.6% of individuals self-identified as of white ethnicity, 5.1% South Asian, 3.7% black, 1.7% not stated, 1.1% other and 0.5% mixed. In 23.3%, ethnicity was missing. 14% of the population experienced polypharmacy—being on five or more medicines, with 3.7% of the population being on 10 or more medicines. The median multimorbidity score in the population was 0.5 (IQR 0–1.15), and the median number of consultations in 2018 was 5 (IQR 1–13) (table 1).

**Table 1:** Demographic descriptive statistics of the study population and the relative frequency of PIP

Variables	Number of individuals—total population (% of population)	Individuals experiencing PIP (% relative frequency of PIP)
Total population (population denominator)	1 200 000 (100)	167 274 (13.9)
Sex*		
Male	612 564 (51.05)	76 177 (12.4)
Female	587 423 (48.95)	91 091 (15.5)
Age		
Mean age (SD)	53.9 (5.58)	55.5 (5.61)
45–49	323 515 (26.96)	31 434 (9.7)
50–54	332 073 (27.67)	40 085 (12.1)
55–59	297 980 (24.83)	45 166 (15.2)
60–64	246 432 (20.54)	50 639 (20.5)
Polypharmacy		
Median (IQR) sum	0 (0–1)	1 (1-2)
0	630 339 (52.5)	10 638 (1.7)
1	160 391 (13.4)	12 054 (7.5)
2–4	240 192 (20.0)	47 991 (20.0)
5–9	124 573 (10.4)	62 035 (49.8)
10+	44 506 (3.7)	34 556 (77.6)
Ethnicity*		
White	774 976 (64.6)	130 864 (16.9)
South Asian	61 294 (5.1)	13 273 (21.7)
Black	43 952 (3.7)	6839 (15.6)
Other	13 582 (1.1)	1491 (11.0)
Mixed	6355 (0.5)	837 (13.2)
Not stated	19 897 (1.7)	2412 (12.2)
Missing	279 944 (23.3)	11 558 (4.1)
**Cambridge Multimorbidity Score**		
Median (IQR)	0.5 (0–1.15)	1.6 (0.92–2.34)
0	415 094 (34.6)	6409 (1.5)
0-<1	466 090 (35.7)	37 128 (8.0)
1-<2	222 381 (18.5)	60 266 (27.1)
2-<4	88 729 (7.4)	57 202 (64.5)
4+	7706 (0.6)	6269 (81.4)
Number of primary care consultations		
Median (IQR)	5 (IQR 1–13)	18 (11–28)
0	291 626 (19.3)	590 (0.2)
1–5	300 630 (25.1)	14 143 (4.7)
5–10	186 085 (15.5)	26 692 (14.3)
10–20	174 440 (14.5)	54 389 (31.2)
20+	79 945 (6.7)	71 460 (89.4)

*13 individuals were intersex, but were not reported due to small numbers, 279 944 individuals had missing ethnicity data. Nb. Due to different population denominators percentages are within columns but not row-wise.

PIP, potentially inappropriate prescribing.

### Prevalence of PIP

PIP prevalence in 2018 was 13.9%. PIP was more common in women compared with men (15.5% vs 12.4%), in older middle-aged adults (60–64 years, 20.5%, 55–59 years, 15.2%, 50–54 years, 12.1%, 45–49 years, 9.7%), in those with South Asian ethnicity (21.7% vs 16.9% white, 15.6% black, 13.2% mixed, 12.2% not stated, 11.0% other), in those with polypharmacy (77.6% in those with 10 or more medicines, 49.8%, 5–9 medicines, 20%, 2–4 medicines, 7.5%, 1 medicine, 1.7%, no medicines at baseline) and those with higher numbers of primary care consultations (89.4% in those with 20 or more consultations, 31.2%, 10–20 consultations, 14.3%, 5–10 consultations, 4.7%, 1–5 consultations, and 0.2%, no consultations) (table 1).

8.9% of the population was exposed to a single PROMPT criterion, with 5% being exposed to multiple criteria (online supplemental table S2). The most common PROMPT exposure was being prescribed a non-steroidal anti-inflammatory drug (NSAID) for longer than 3 months (7.07%) and long-term high-dose proton pump inhibitors 3.02% (table 2).

**Table 2:** Number and proportion of population experiencing each of the PROMPT potentially inappropriate prescribing criteria

Prompt criteria	Number of patients experiencing prompt in 2018 (% experiencing individual criteria)
Other than for opioid induced constipation, stimulant laxatives should not be prescribed as first-line treatment in constipation for greater than 4 weeks.	4894 (0.41)
Proton pump inhibitors (PPIs) (eg, esomeprazole, omeprazole) should not be prescribed at doses above the recommended maintenance dosage for greater than 8 weeks.	36 260 (3.02)
Esomeprazole or omeprazole should not be used in combination with clopidogrel.	<10 (<0.01)
The use of alpha-adrenoreceptor blocking drugs as monotherapy for hypertension should be avoided.	1131 (0.94)
Aspirin doses should not exceed 150 mg/day for antiplatelet therapy.	770 (0.06)
Cardioselective CCB (Verapamil and Diltiazem) should not be used in combination with beta-adrenoceptor drugs.	741 (0.06)
The use of oral-short acting dipyridamole should not be used as monotherapy in antiplatelet therapy.	18 (<0.01)
First generation antihistamines should not be used as first-line agents for greater than 7 days	17 206 (1.43)
Theophylline should not be used as monotherapy for asthma or chronic obstructive pulmonary disease.	226 (0.02)
A concomitant bisphosphonate should be prescribed if oral corticosteroids are used long term (greater than 3 months).	10 442 (0.87)
Mucolytic agents should not be used in stable chronic COPD.	676 (0.05)
SSRIs should not be used in combination with venlafaxine.	<10 (<0.01)
Tricyclic antidepressants should not be used as first line in treatment of depression.	10 518 (0.88)
Benzodiazepines (e.g. nitrazepam, temazepam) should not be used long-term (greater than 4 weeks).	10 719 (0.89)
Non-benzodiazepine hypnotics should not be used long-term (greater than 4 weeks).	9775 (0.81)
Carbamazepine should not be used in combination with clarithromycin or erythromycin.	48 (<0.01)
Strong opioids (e.g. buprenorphine, diamorphine, fentanyl, morphine, oxycodone) should not be prescribed without the co-prescribing of laxatives.	27 404 (2.28)
Nitrofurantoin should not be prescribed for greater than 7 days for the management of uncomplicated lower UTIs	3081 (0.26)
In relation to the management of diabetes, the use of glibenclamide should be avoided.	41 (<0.01)
NSAIDs should not be used for greater than 3 months.	84 798 (7.07)
Unless adequate GI protection is provided with either a PPI or H2 receptor antagonist, NSAIDs should not be used in combo with low dose aspirin or SSRIs	21 372 (1.78)
The use of two or more drugs from the same pharmacological class should be avoided, unless used for additive effects in line with clinical guidelines.	21 822 (1.82)

CCB, Calcium channel blocker; COPD, Chronic obstructive pulmonary disease ; GI, Gastro-intestinal; NSAIDs, Non-steroidal anti-inflammatory drugs; PROMPT, Prescribing Optimally in Middle-aged People’s Treatments; SSRIs, Selective serotonin re-uptake inhibitors; UTIs, Urinary tract infections.

### Demographic factors associated with PIP

Demographic factors associated with greater odds of exposure to PIP were increasing age (OR-1.01, CI 1.01 to 1.02), being of South Asian (OR-1.10, CI 1.09 to 1.11) or black ethnicity (OR-1.05, CI 1.05 to 1.08) compared with white ethnicity, polypharmacy (2–4 medications, OR-1.80 CI 1.77 to 1.83, with 5–9 medications OR-5.00, CI 4.90 to 5.11, with 10+ medications OR-6.93 CI 6.61 to 7.27), number of primary care consultations during the exposure year (OR-1.04, CI 1.04 to 1.04), and having a higher Cambridge multimorbidity score (OR-2.47, CI 2.45 to 2.50). Compared with women, men had lower odds of receiving PIP (OR-0.87, CI 0.86 to 0.88), as did people of other ethnicity (OR-0.90, CI 0.89 to 0.92), or not-stated ethnicity (OR-0.90, CI 0.89 to 0.92). The intraclass correlation was low (ICC-0.03, CI 0.03 to 0.03), indicating that the proportion of PIP explained by GP practice was small (table 3). A Poisson model (sensitivity analysis) showed similar associations (online supplemental table S3).

**Table 3:** Association of demographic factors with potentially inappropriate prescribing, partially and fully adjusted multi-level logistic regression*

	OR (partially adjusted)	95% CI	OR (fully-adjusted)	95% CI	P value
Sex					
Female	Ref		Ref		
Male	0.77	0.76 to 0.78	0.87	0.86 to 0.88	<0.001
Age (years)	1.06	1.06 to 1.06	1.01	1.01 to 1.02	<0.001
Ethnicity					
White	Ref		Ref		
South Asian	1.47	1.44 to 1.50	1.10	1.09 to 1.11	<0.001
Black	0.97	0.95 to 1.00	1.05	1.05 to 1.08	<0.001
Mixed	0.82	0.76 to 0.88	0.99	0.97 to 1.01	0.258
Other	0.65	0.62 to 0.69	0.90	0.89 to 0.92	<0.001
Not stated	0.71	0.68 to 0.74	0.90	0.89 to 0.92	<0.001
Polypharmacy					
0–1	Ref		Ref		
2–4	3.66	3.61 to 3.72	1.80	1.77 to 1.83	<0.001
5–9	29.5	29.04 to 30.03	5.00	4.90 to 5.11	<0.001
10+	122.4	117.48 to 127.52	6.93	6.61 to 7.27	<0.001
Cambridge Multimorbidity Score	4.89	4.86 to 4.93	2.47	2.45 to 2.50	<0.001
Number of consultations (2018)	1.10	1.10 to 1.10	1.04	1.04 to 1.04	<0.001

* Model partially adjusted age and sex. Fully adjusted for variation between practices, age, sex, polypharmacy, multimorbidity, ethnicity.

### Associations between PIP and number of consultations

After adjusting for hypothesised confounders, PIP exposure in 2018 was associated with an increased rate of primary care consultations the following year (incident rate ratio IRR 1.03, CI 1.02 to 1.03) (table 4).

**Table 4:** Association of potentially inappropriate prescribing with primary care consultations and mortality.Partially and fully adjusted negative binomial regression to examine consultations. Partially and fully adjusted cox-regression for mortality*

	Incident rate ratio (partially-adjusted)	95% CI	P value	Incident rate ratio (fully-adjusted)	95% CI	P value
Number of consultations in 2019	2.67	2.51 to 262	<0.001	1.03	1.02 to 1.03	<0.001
Mortality	2.61	2.51 to 2.72	<0.001	0.99	0.93 to 1.04	0.601

* models partially adjusted for age and sex. Fully adjusted for variation between practices, age, sex, polypharmacy, multimorbidity, ethnicity, consultation frequency in 2018

### Associations between PIP and mortality

151 individuals were excluded as they deregistered or died before the end of the exposure period. A total of 4 675 481 years of at-risk time were observed with 12 572 deaths between 1 January 2019 and 7 February 2023. After adjusting for hypothesised confounders, there was no evidence for a relationship between PIP exposure in 2018 and subsequent mortality (HR 0.99, CI 0.93 to 1.04) (table 4)

### Sensitivity analysis

Sensitivity analysis with PIP as a count rather than binary variable showed no significant difference in associations detected (online supplemental table S3). Complete case analysis identified the same associations (online supplemental table S5) and removing outliers did not significantly change the results (online supplemental table S5). Limiting follow-up of mortality to 2019 did not alter the significance of association between PIP and mortality (online supplemental table S6).

## Discussion

In this nationally representative cohort of 1.2 million UK adults aged 45–64 years, approximately one in seven were exposed to PIP. PIP was associated with female sex, increasing age, polypharmacy, multimorbidity and frequent primary care consultations. We observed elevated odds of PIP among individuals from South Asian and Black ethnic backgrounds. While PIP was not associated with mortality, it predicted a modest but significant increase in subsequent consultation rates, suggesting a burden of potentially avoidable healthcare use.

### Comparison with the existing literature

The prevalence of PIP was lower in middle-aged adults than in older adults (typically 29%).^38^ Differences from previous UK studies—such as one reporting 19%^20^ prevalence in a highly deprived, urban cohort—may reflect variations in population structure and care access.^39 40^ Our findings reinforce established associations between PIP and female sex, older age within the cohort, and higher treatment burden, consistent with prior UK and Irish studies.^20 24^

The novel finding of higher PIP risk among South Asian and Black individuals contrasts with US Medicare data, which report lower rates among ethnic minority groups.^41^ These discrepancies may reflect differences in healthcare systems, medication use patterns and sociocultural influences. The role of ethnic disparities in medication safety warrants further investigation, particularly given concerns around equitable access and treatment burden.

The association between PIP subsequent consultation rates—though modest—indicates that PIP may contribute to a cycle of increased healthcare demand, even in younger age groups. Previous studies were underpowered to detect this relationship.^24^ The absence of an association between PIP and mortality is not unexpected, given the lower baseline risk in mid-life and the short follow-up period for long-term outcomes.

### Strengths and limitations

The study’s strengths include its scale, national representativeness and use of validated PROMPT criteria tailored to middle-aged adults. The robust analytic approach, including multilevel modelling and multiple imputation for missing ethnicity data, supports the reliability of findings.

The absence of linked secondary care and socioeconomic data limits our ability to fully adjust for all known confounding factors. Mortality data limited to primary care records may underestimate true mortality rates, potentially causing misclassification bias.^42^ Although, as CPRD mortality data have previously been shown to be relatively complete any effect is not likely to change our findings. Additionally, PROMPT criteria represent a composite measure, preventing analysis of individual prescribing scenarios most strongly associated with adverse outcomes. The fact that 23.3% of data on patient ethnicity was missing and imputed means that the associations of PIP with ethnicity should be interpreted with caution.

There is the risk of information bias because of variability of recording of data.

There is likely to be residual confounding particularly from previous consultation data not fully characterising baseline health usage/demand, and from patient deprivation. Although this may be partially accounted for by the models adjusting for practice-practice variation. CPRD only captures primary care prescribing and adherence cannot be measured, so there will be some misclassification of PIP.

### Clinical and policy implications

There are 17.4 million middle-aged adults in the UK. With 13.9% of these adults experiencing PIP and there being an associated 3% increase in GP consultations, this would amount to an extra 362 790 consultations a year (~one in every thousand GP appointments being attributable to PIP in middle-aged adults). Although at an individual level, this increased utilisation may not be clinically significant, at a population level, it is a significant increase in demand on a stretched primary healthcare system.

Randomised trials suggest that structured medication reviews, informed by explicit criteria, reduce adverse drug events and healthcare utilisation.^43^ Despite this, structured reviews in UK general practice are largely restricted to adults aged ≥65 or those in care homes.^44 45^ Our findings suggest that extending these reviews to high-risk middle-aged adults should be explored to see if it has the potential to reduce inappropriate prescribing and primary care usage.

PROMPT criteria could be embedded into electronic prescribing systems, enabling identification of suboptimal prescribing—potentially reducing overprescribing and improving patient safety.

### Future research directions

Further research is needed to identify which PROMPT criteria are most strongly associated with adverse outcomes. Linking to hospitalisation and prescribing cost data would enable fuller economic evaluation. Qualitative studies exploring the experiences of patients and prescribers could inform person-centred deprescribing interventions. Finally, randomised trials are required to assess the effectiveness and feasibility of PROMPT-informed structured medication reviews in middle-aged adults.

## Conclusion

This study highlights a substantial burden of PIP in UK adults aged 45–64, particularly among those with multimorbidity, polypharmacy and from ethnic minority backgrounds. Our findings suggest that structured medication review strategies should be evaluated for at-risk middle-aged populations. PROMPT criteria offer a scalable, evidence-based tool for targeting prescribing improvements in middle age. Embedding such tools within general practice systems may reduce avoidable healthcare use and support safer prescribing.

## Supplementary material

10.1136/bmjopen-2025-115606 online supplemental table 1

## Data Availability

Data may be obtained from a third party and are not publicly available.
