# Supplementary material for: Is potentially inappropriate prescribing in UK middle-aged adults associated with healthcare utilisation and mortality? A prospective cohort study of a 1.2 million patient cohort conducted in the clinical practice research datalink
Source: BMJ Open. 2026 Jul 27;16(7):e115606. doi: 10.1136/bmjopen-2025-115606 (PMC13409091; doi:10.1136/bmjopen-2025-115606)
Supplement: online supplemental table 1 [file bmjopen-16-7-s001.docx]

### Supplementary Tables:

#### **Table S1: PROMPT criteria for middle aged adults and how this was defined in CPRD between the dates 1^st^ January 2018 and 31^st^ December 2018**

| **PROMPT Criterion** | **Extraction and criteria construction notes** |
| --- | --- |
| 1. Other than for opioid-induced constipation, stimulant laxatives should not be prescribed as first-line treatment in constipation for greater than  4 weeks. | - Exclude patients with opioid prescription. - Exclude patients with a stimulant laxative prescription and ≥ 1 other laxative. - Include patients only prescribed a stimulant laxative: 2 or more scripts within 60 days or 3 or more in 6 months. |
| 2. Proton pump inhibitors (PPIs) (e.g. esomeprazole, omeprazole) should not be prescribed at doses above the recommended maintenance dosage for greater than eight weeks. | - Include those with high dosage prescriptions: 40mg omeprazole, 40mg esomeprazole, 40mg pantoprazole, 20mg rabeprazole, 30mg lansoprazole. - Number of patients with 2 or more high-dosage PPI scripts within 60 days, or 3 or more in 6 months. |
| 3. Esomeprazole or omeprazole should not be used in combination with clopidogrel. | - Number of patients with esomeprazole/omeprazole and clopidogrel prescriptions within same month. |
| 4. The use of alpha-adrenoreceptor blocking drugs as monotherapy for hypertension should be avoided. | - Number of patients with hypertension diagnosis prescribed an alpha-adrenoreceptor blocker after diagnosis, without an ACE-inhibitor , ARB (angiotensin receptor blocker), CCB (Calcium Channel Blocker), thiazide diuretic or beta-blocker. |
| 5. Aspirin doses should not exceed 150mg/day for antiplatelet therapy. | - Number of patients prescribed Aspirin 300mg scripts 2 or more in 6 months. ***Assume that all aspirin prescriptions are for anti-platelet therapy.*** |
| 6. Cardio-selective CCB (Verapamil and Diltiazem) should not be used in combination with betaadrenoceptor drugs. | - Number of patients prescribed verapamil or diltiazem and a betablocker within same month. |
| 7. The use of oral-short acting dipyridamole should not be used as monotherapy in antiplatelet therapy. | - Number of patients prescribed dipyridamole without any other antiplatelet medicine in the same month. ***Assume that dipyridamole only used for antiplatelet therapy.*** |
| 8. First generation antihistamines should not be used as first-line agents for greater than 7 days. | - Number of patients prescribed first generation anti-histamines for more than 7 days. - Of those patients, exclude if they have been prescribed any other non-first-generation antihistamine/allergen immunotherapy. |
| 9. Theophylline should not be used as monotherapy for asthma or chronic obstructive pulmonary disease. | - Number of patients with theophylline prescription without any other asthma/COPD treatment (antimuscarinics, bronchodilators, corticosteroids, leukotriene receptor antagonists, mucolytics). |
| 10. A concomitant bisphosphonate should be prescribed if oral corticosteroids are used long term (greater than three months). | - Patients with corticosteroid prescriptions 3 or more in 6 months without a bisphosphonate prescription in the same six-month period. |
| 11. Mucolytic agents should not be used in stable chronic COPD. | - Number of patients with mucolytic prescription and COPD diagnosis without any oral corticosteroid script **(defined as stable**  **COPD if not on oral corticosteroid)**. |
| 12. SSRIs should not be used in combination with venlafaxine. | Number of patients with SSRI prescription and venlafaxine within same month. |
| 13. Tricyclic antidepressants should not be used as first-line in treatment of depression. | Number of patients with depression diagnosis and a tricyclic prescription before a monoamine oxidase inhibitor, SSRI or other antidepressant prescription. (all historic antidepressant prescriptions extracted and used) |
| 14. Benzodiazepines (e.g. nitrazepam, temazepam) should not be used long-term (greater than four weeks). | Number of patients with 2 or more benzodiazepine scripts within 3 months or 3 or more in 6 months. |
| 15. Non-benzodiazepine hypnotics should not be used long-term (greater than 4 weeks). | Number of patients with 2 or more non-benzodiazepine hypnotic scripts within 3 months or 3 or more in 6 months. |
| 16. Carbamazepine should not be used in combination with clarithromycin or erythromycin. | -Number of patients with carbamazepine prescription and that are prescribed clarithromycin or erythromycin within same month. |
| 17. Strong opioids (e.g. buprenorphine, diamorphine, fentanyl, morphine, oxycodone) should not be prescribed without the co-prescribing of laxatives. | -Number of patients with 3 or more strong opioid scripts within 6 months.  -Exclude those prescribed a laxative within this 6-month period. |
| 18. Nitrofurantoin should not be prescribed for greater than 7 days for the management of uncomplicated lower UTIs. | -Number of patients prescribed nitrofurantoin for more than one week.  ***-Assume nitrofurantoin given for uncomplicated UTIs*** |
| 19. In relation to the management of diabetes, the use of glibenclamide should be avoided. | -Number of patients prescribed glibenclamide and with a diabetes diagnosis. |
| 20. NSAIDs should not be used for greater than 3 months. | -Number of patients with NSAIDs script 3 or more prescriptions in 6 months. |
| 21. Unless adequate GI protection is provided with either a PPI or H_2_ receptor antagonist, NSAIDs should not be used in combo with low dose aspirin or SSRIs. | Number of patients with NSAIDs prescription without PPIs or H2 receptor antagonist prescription within the same month.  Of those patients, the number co-prescribed SSRIs or aspirin. |
| 22. The use of two or more drugs from the same pharmacological class should be avoided, unless used for additive effects in line with clinical guidelines. | Patients with 3 prescriptions within 6 months of one drug from any of the classes below. Of these patients, look for a different drug prescribed from the same class within same 6-month window. i.e. greater than or equal to 2 z drug hypnotics, greater than or equal to 2 benzodiazepines, greater than or equal to 2 opioids scripts in 6 months etc.  Drug classes: Non-benzodiazepine Z drug hypnotics,  Benzodiazepines , Opioids, Loop diuretics, NSAIDs, ACE inhibitors,  ARBs, SSRIs, TCAs, CCBs, Beta Blockers, Statins, Thiazide Diuretics. |

**Table S2: Exposure to PROMPT criteria within the study population**

| Number of prompt criteria population exposed to in 2018 | Number of patients (% of population) |
| --- | --- |
| 0 | 1,032,726 (86.1) |
| 1 | 107,217 (8.9) |
| 2 | 36,318 (3.0) |
| 3 | 16,167 (1.3) |
| 4 | 5,530 (0.5) |
| 5 | 1,569 (0.1) |
| 6 | 375 (<0.1) |
| 7 | 82 (<0.1) |
| 8 | 15 (<0.1) |
| 9 | 1(<0.1) |
| Any prompt criteria | 167,274 (13.9) |

|  | **Incident rate ratio (fully-adjusted)** | **95% confidence interval** | **P-value** |
| --- | --- | --- | --- |
| **Sex** |  |  |  |
| Female | Ref |  |  |
| Male | 0.91 | 0.90-0.92 | **<0.001** |
| **Age (years)** | 1.01 | 1.01-1.01 | **<0.001** |
| **Ethnicity** |  |  |  |
| White | Ref |  |  |
| South Asian | 1.07 | 1.05-1.09 | **<0.001** |
| Black | 0.96 | 0.94-0.99 | **0.001** |
| Mixed | 0.98 | 0.93-1.04 | 0.465 |
| Other | 0.96 | 0.92-1.00 | 0.039 |
| Not stated | 0.99 | 0.96-1.03 | 0.699 |
| **Polypharmacy** |  |  |  |
| 0-1 | Ref |  |  |
| 2-4 | 1.88 | 1.86-1.91 | **<0.001** |
| 5-9 | 5.59 | 5.52-5.67 | **<0.001** |
| 10+ | 5.85 | 5.74-5.96 | **<0.001** |
| **Cambridge multimorbidity score** | 1.45 | 1.44-1.45 | **<0.001** |
| **Number of consultations (2018)** | 1.01 | 1.01-1.01 | **<0.001** |

**Table S3: Association of demographic factors with number of potentially inappropriate prescribing exposures. A fully adjusted multi-level Poisson regression (adjusted for variation between practices, age, sex, polypharmacy, multimorbidity, ethnicity)**

Table S4: Complete case analysis **Association of potentially inappropriate prescribing with primary care consultations and mortality. Partially and fully adjusted negative binomial regression to examine consultations. Partially and fully adjusted cox-regression for mortality*.**

|  | **Incident-rate ratio (Partially-adjusted)** | **95% confidence interval** | **p-value** | **Incident rate ratio (fully-adjusted)** | **95% confidence interval** | **p-value** |
| --- | --- | --- | --- | --- | --- | --- |
| **Number of consultations in 2019** | 2.68 | 2.64-2.71 | <0.001 | 1.05 | 1.04-1.05 | **<0.001** |
| **Mortality** | 2.55 | 2.36-2.75 | <0.001 | 1.04 | 0.94-1.15 | 0.394 |

*** models partially adjusted for age and sex. Model fully adjusted for variation between practices, age, sex, polypharmacy, multimorbidity, ethnicity, consultation frequency in 2018 .**

S5: **Association of potentially inappropriate prescribing with primary care consultations and mortality. Partially and fully adjusted negative binomial regression to examine consultations. Partially and fully adjusted cox-regression for mortality*. Excluding those with high consultation rates (>20/year in 2019) :**

|  | **Incident-rate ratio (Partially-adjusted)** | **95% confidence interval** | **p-value** | **Incident rate ratio (fully-adjusted)** | **95% confidence interval** | **p-value** |
| --- | --- | --- | --- | --- | --- | --- |
| **Number of consultations in 2019** | 2.68 | 2.64-2.71 | <0.001 | 1.05 | 1.04-1.05 | **<0.001** |
| **Mortality** | 2.55 | 2.36-2.75 | <0.001 | 1.04 | 0.94-1.15 | 0.394 |

|  | **Incident-rate ratio (Partially-adjusted)** | **95% confidence interval** | **p-value** | **Incident rate ratio (fully-adjusted)** | **95% confidence interval** | **p-value** |
| --- | --- | --- | --- | --- | --- | --- |
| Number of consultations in 2019 | 2.03 | 2.02-2.05 | <0.001 | 1.04 | 1.03-1.04 | <0.001 |
| Mortality | 1.90 | 1.78-2.02 | <0.001 | 1.04 | 0.96-1.14 | 0.329 |

*** models partially adjusted for age and sex. Model fully adjusted for variation between practices, age, sex, polypharmacy, multimorbidity, ethnicity, consultation frequency in 2018 .**

**S6: Association or potentially inappropriate prescribing with mortality a fully adjusted cox-regression model, with restriction to one year post exposure (2019). Model fully adjusted for variation between practices, age, sex, polypharmacy, multimorbidity, ethnicity, and consultation frequency in 2018.**

|  | Incident rate ratio fully- adjusted | 95% Confidence interval | p-value |
| --- | --- | --- | --- |
| Mortality | 1.03 | 0.76-1.38 | 0.871 |
