# Supplementary material for: Is potentially inappropriate prescribing in UK middle-aged adults associated with healthcare utilisation and mortality? A prospective cohort study of a 1.2 million patient cohort conducted in the clinical practice research datalink
Source: BMJ Open. 2026 Jul 27;16(7):e115606. doi: 10.1136/bmjopen-2025-115606 (PMC13409091; doi:10.1136/bmjopen-2025-115606)
Supplement: Supplementary file 2 [file bmjopen-2025-115606.reviewer_comments.pdf]

PEER REVIEW HISTORY

BMJ Open publishes all reviews undertaken for accepted manuscripts. Reviewers are asked to complete a checklist review form and are provided with free text boxes to elaborate on their assessment. These free text comments are reproduced below.

ARTICLE DETAILS

Title (Provisional)

Is Potentially inappropriate prescribing in UK middle-aged adults associated with healthcare utilisation and mortality? A prospective cohort study of a 1.2 million patients conducted in the Clinical Practice Research Datalink

Authors

Naughton, Michael; Molokhia, Mariam; Redmond, Patrick; Douglas, Ian J; Mathur, Rohini

---

VERSION 1 - REVIEW

**Reviewer**	**1**
**Name**	**mitchell, aaron**
**Affiliation**	**Memorial Sloan Kettering Cancer Center**
**Date**	**08-Feb-2026**
**COI**	**None**

This study of potentially inappropriate prescribing has a number of strengths. Chief among them is the significance of this problem – PIP can lead to medical harms on the patient level and contributes to wasteful spending and high health care costs on the system level. The population-level design and large sample size are also strengths. The epidemiologic and statistical approach are largely sound, and the authors have given attention to a number of the key sources of bias and confounding, such as practice-level correlation and visit intensity in the baseline period. Table S1 is very helpful in terms of explaining and documenting the methodology. Mitigating the overall significance is the fact that PIP has already been studied in the older age group where risks are highest.

In terms of limitations and recommendations for improvement, I have notice several major limitations and a few smaller details:

- Major: The sensitivity analyses are well-considered and appropriately selected. But their results should be presented; suggest addition of supplementary tables with these results.

- Major: A few of the forms of PIP seem unbelievably rare, raising concern for issues with data capture in the CPRD. For example, concurrent clopidogrel and PPI. These are both very

common medications, and it is hard to believe that this never happens while (for counterexample) excessive PPI durations were observed over 36,000 times. What is the potential explanation? Might it be that clopidogrel is limited to cardiology specialists in the UK system, such that these prescriptions do occur but are not observed?

- Minor: The investigators note that death data is from the CPRD and may be incomplete. This is a potentially large concern given that death is a primary outcome, and one could imagine selection effects which might result in deaths being unobserved in this data source. How well do death rates using CRPC data correlated with population averages – do they line up or does CRPC substantially undercount? The authors cite a prior work (#42) which appears to show that CPRD is in fact very accurate; if the authors agree with this interpretation then it would be reasonable to state this in the discussion and that the magnitude of this bias is likely to be small.

- Minor: the manuscript mentions “residual confounding” as a potential limitation. This is true, but it would be more helpful to the lay reader what kind of confounders might remain, so that they can better judge the potential importance. The potential residual confounder that most stood out to me in reading the article is the underlying characteristic of health care need in 2018. The authors adjust for this quantity by measuring consultations in 2018. This measurement approach may group together patients who are quite different in their 2019 health care needs – consider a patient who had 2 well checkups in March and October 2018, vs. a patient who had two PCP visits for new onset back pain in December 2018. The timing of the visits might therefore correlate both with the likelihood of PIP (e.g., excessive NSAID prescriptions) and the likelihood of continued PCP visits at above-baseline frequency in 2019 related to the new symptom.

- Minor: The manuscript describes categorizations of “white” and “black” as ethnicity, but I believe these are typically considered racial categories (in that they are applied based on physical traits, as opposed to cultural or national background).

**Reviewer**	**2**
**Name**	**Dack, Kyle**
**Affiliation**	**University of Bristol, MRC Integrative Epidemiology Unit**
**Date**	**10-Feb-2026**
**COI**	**None**

Statistical review summary:

The design and analysis are reasonable, and the conclusions are supported by the data. I have two main suggestions which are (a) it is unclear how the 1.2 random sample was arrived at, and (b) given the size of the analysis, significance testing loses some of its value as it may be hard to find variables not significance. This does not undermine the interpretation

but it would be beneficial to add more interpretation of the actual effect sizes, their clinical significance, and discuss in more detail whether unmeasured confounding (SES) would be enough to account for these effect sizes.

Abstract:

1. Overall the abstract is clear, containing all important methodological information. I suggest also adding the N either to the participants or results section.

Methods:

2. Study participants – the 1.2 million random sample is out of how many in total? What was the rationale for this specific number to sample?

3. Exposure: For readers unfamiliar with the data source it may be helpful to clarify how the PROMPT data was generated, e.g. an automatic evaluation of patient disease and prescription parameters within CPRD?

4. "for PIPs association with outcomes they were confounders" I suggest hedging this as "potential confounders" or similar, as there appears to be uncertain evidence that they do affect PIP (hence described as potential explanatory factors in the cross-sectional model).

5. Imputation: Could more information be added regarding the imputation – the package/function, and parameters (imputations, iterations)?

6. Multilevel models: the models are clearly specified, and rationale is provided for the key choices. Overall the analysis looks reasonable and I have no concerns. However, the means by which clustering is accounted for is not specified, e.g. was a random intercept or random slope added, and what was the rationale for this?

Results:

7. "Interaction between polypharmacy and multimorbidity was found to be significant in all models, so it was retained." I could not follow this sentence, it seems somewhat out of place in the descriptive statistics. If involves the interaction term being retained as part of the modelling strategy, it may be more suitable at the beginning of the model section? However, having looked at the model tables, there does not appear to be any interactions, and this is the final mention of interactions in the manuscript, so I am unsure.

8. Table 1. There is a risk that readers may try to compare the percentages between column 1 and 2, but the denominators are different. The column labels already report this but it could be missed if readers jump to a specific statistic. I suggest considering an in-text note to explain table 1, or a footnote to reiterate that percentages are not comparable row-wise.

9. Model results: It would be helpful to re-iterate the statistical model applied before getting into the results, e.g. a single sentence reminding readers of what was modelled on what, and to confirm the N (I assume 1.2 million if imputation was used, but it would be clearer to confirm directly in-text and/or in the relevant tables.

10. Table 3: There is an incomplete parenthesis in the footnote.

11. Sensitivity analyses: This section seems weakly supported as no tables of results are provided. Additionally, it is unclear if any of the reported findings relate to sensitivity analysis 4 (mortality restricted to 2019 to avoid Covid-19). It would significantly strengthen this section if supplementary tables could be provided.

Discussion:

12. The discussion is supported by the data, the key caveats are noted and the conclusions valid. However, the study has extremely high statistical power which means that significance testing is less informative, and I notice that the effect size or clinical significance of the effect estimates is not commented on. Adding some comment on effect magnitude could add more detail to the interpretation.

VERSION 1 - AUTHOR RESPONSE

Reviewer: 1
Dr. Aaron Mitchell, Memorial Sloan Kettering Cancer Center
Comments to the Author:
This study of potentially inappropriate prescribing has a number of strengths. Chief among them is the significance of this problem – PIP can lead to medical harms on the patient level and contributes to wasteful spending and high health care costs on the system level. The population-level design and large sample size are also strengths. The epidemiologic and statistical approach are largely sound, and the authors have given attention to a number of the key sources of bias and confounding, such as practice-level correlation and visit intensity in the baseline period. Table S1 is very helpful in terms of explaining and documenting the methodology. Mitigating the overall significance is the fact that PIP has already been studied in the older age group where risks are highest.

In terms of limitations and recommendations for improvement, I have notice several major limitations and a few smaller details:

- Major: The sensitivity analyses are well-considered and appropriately selected. But their results should be presented; suggest addition of supplementary tables with these results.

Thank you for highlighting this. The results from the sensitivity analyses have been added in supplementary tables S3, S4, S5, and S6.

- Major: A few of the forms of PIP seem unbelievably rare, raising concern for issues with data capture in the CPRD. For example, concurrent clopidogrel and PPI. These are both very common medications, and it is hard to believe that this never happens while (for counterexample) excessive PPI durations were observed over 36,000 times. What is the potential explanation? Might it be that clopidogrel is limited to cardiology specialists in the UK system, such that these prescriptions do occur but are not observed?

Thank you for highlighting this, we noted this during analysis and had the same concern. We went back to check the drug codes for clopidogrel and re-ran searches for clopidogrel in CPRD, this didn't identify any issues. We also cross checked the CPRD code list against the university of Manchester's clinicalcodes.org. Although there was some clopidogrel prescribing in our group it was rare, and even rarer for it to be co-prescribed with omeprazole.

We used the same code list as a previous study, Khatter el al., that examined the PROMPT criteria prescribing in a very deprived London population and who only found 0.15% prevalence of clopidogrel/omeprazole co-prescribing. Although ours was lower still (by a factor of 10). We thought the following reasons would mean that there truly is a lower prevalence of this indicator in this group.

We thought of the following possible reasons:

1) **NICE guidance for ACS had changed by the time of our study to recommend Ticacgrelor rather than clopidogrel prescription. This has led to a much lower levels of Clopidogrel prescribing.**

2) GP systems such as Emis had introduced a severe drug warning for the omeprazole and clopidogrel combination making them unlikely to be co-prescribed in primary care. Other PPIs such as Lansoprazole would be prescribed instead.
3) This group is young and has a low prevalence of stroke or ACS, so anticoagulant prescribing prevalence is very low anyway.
4) Our sample being nationally representative, it is likely to be less deprived than the population examined in Khatter et al, so lower rates of stroke and ACS, and thus less anticoagulation.
5) Nationally some of the prescribing may be undertaken in secondary care and so not captured.

In my locality Ticagrelor and clopidogrel prescribing are taken over by primary care, although perhaps this varies by region.

For the other rare PROMPT criteria, the level we found were similar to levels found in previous studies and we were not concerned as the drugs are much less likely to be prescribed.

- Minor: The investigators note that death data is from the CPRD and may be incomplete. This is a potentially large concern given that death is a primary outcome, and one could imagine selection effects which might result in deaths being unobserved in this data source. How well do death rates using CRPC data correlated with population averages – do they line up or does CRPC substantially undercount? The authors cite a prior work (#42) which appears to show that CPRD is in fact very accurate; if the authors agree with this interpretation then it would be reasonable to state this in the discussion and that the magnitude of this bias is likely to be small.

Thank you for highlighting this, we have changed the statement, the death rates were similar to population averages. Due to this and the previous work we felt any bias is likely to be small but still wanted to acknowledge it as a limitation. Due to death rates not even suggesting a difference we think that any minor bias is extremely unlikely to alter the result.

- Minor: the manuscript mentions "residual confounding" as a potential limitation. This is true, but it would be more helpful to the lay reader what kind of confounders might remain, so that they can better judge the potential importance. The potential residual confounder that most stood out to me in reading the article is the underlying characteristic of health care need in 2018. The authors adjust for this quantity by measuring consultations in 2018. This measurement approach may group together patients who are quite different in their 2019 health care needs – consider a patient who had 2 well checkups in March and October 2018, vs. a patient who had two PCP visits for new onset back pain in December 2018. The timing of the visits might therefore correlate both with the likelihood of PIP (e.g., excessive NSAID prescriptions) and the likelihood of continued PCP visits at above-baseline frequency in 2019 related to the new symptom.

We agree with your assessment, we have added to our statement on residual confounding to highlight where the main limitations due to this lie.

"There is likely to be residual confounding particularly from previous consultation data not fully characterising baseline health usage/demand and from patient deprivation."

- Minor: The manuscript describes categorizations of "white" and "black" as ethnicity, but I believe these are typically considered racial categories (in that they are applied based on physical traits, as opposed to cultural or national background).

Ethnicity groupings were used that had previously been applied for OpenSafely Research, they are based on ethnicity as self-reported and recorded within the GP records. We acknowledge that the groupings are broad and there is going to be significant heterogeneity within the categorises, including due to differing countries of origin. The categorises were aggregated to enable analysis. However, using a larger number of categorises with smaller numbers within them was considered difficult to interpret and less feasible.

Reviewer: 2
Dr. Kyle Dack, University of Bristol
Comments to the Author:
Statistical review summary:
The design and analysis are reasonable, and the conclusions are supported by the data. I have two

main suggestions which are (a) it is unclear how the 1.2 random sample was arrived at, and (b) given the size of the analysis, significance testing loses some of its value as it may be hard to find variables not significance. This does not undermine the interpretation but it would be beneficial to add more interpretation of the actual effect sizes, their clinical significance, and discuss in more detail whether unmeasured confounding (SES) would be enough to account for these effect sizes.

Abstract:
1. Overall the abstract is clear, containing all important methodological information. I suggest also adding the N either to the participants or results section.
I have added this to the participants section of the abstract.

Methods:
2. Study participants – the 1.2 million random sample is out of how many in total? What was the rationale for this specific number to sample?

A sentence to clarify this has been added. A sample size calculation was carried out for the study to have power to detect a difference in mortality as detailed below. Sample size was rounded up to a round 100,000.
"The population size was selected to have a >80% power to detect a 10% difference in mortality (p<0.05), as the most important but also rarest outcome."

3. Exposure: For readers unfamiliar with the data source it may be helpful to clarify how the PROMPT data was generated, e.g. an automatic evaluation of patient disease and prescription parameters within CPRD?

Thank you for raiding this, we have added further detail and readers are directed to supplementary table S1 if they want to see the detail of how each PIP criteria was constructed. We hope this makes the paper clearer for readers.
"These were constructed using issued prescription data and diagnosis codes extracted from CPRD."

4. "for PIPs association with outcomes they were confounders" I suggest hedging this as "potential confounders" or similar, as there appears to be uncertain evidence that they do affect PIP (hence described as potential explanatory factors in the cross-sectional model).

Wording has been changed to potential confounders in the methods section.

5. Imputation: Could more information be added regarding the imputation – the package/function, and parameters (imputations, iterations)?

Thank you for highlighting this, we have added detail and hopes this further clarifies how values were imputed.

"Multiple imputation by chained equations addressed missing ethnicity data [37], using multivariate method with multinominal logistic regression and 10 iterations"

Stata's mi impute chained command was used using default specification of the prediction equations

6. Multilevel models: the models are clearly specified, and rationale is provided for the key choices. Overall the analysis looks reasonable and I have no concerns. However, the means by which clustering is accounted for is not specified, e.g. was a random intercept or random slope added, and what was the rationale for this?

Detail added to the statistical analysis section:
"Multi-level models were used with patients grouped by practice using a random intercept model, to allow for practice to practice difference in clinician prescribing practices."

The rationale was that different practices may have different prescribing habits/cultures/preferences. But that the effect of the other covariates would not vary by practice, thus no random slope.

Results:
7. "Interaction between polypharmacy and multimorbidity was found to be significant in all models, so it was retained." I could not follow this sentence, it seems somewhat out of place in the descriptive statistics. If involves the interaction term being retained as part of the modelling strategy, it may be more suitable at the beginning of the model section? However, having looked at

the model tables, there does not appear to be any interactions, and this is the final mention of interactions in the manuscript, so I am unsure.

Sentence is an error from copied from my previous draft for my master's dissertation. Final logistic regression model didn't have an interaction term, thus not reported in table. I have removed the sentence. Table 4 is a causal model, so covariates are not reported here.

8. Table 1. There is a risk that readers may try to compare the percentages between column 1 and 2, but the denominators are different. The column labels already report this but it could be missed if readers jump to a specific statistic. I suggest considering an in-text note to explain table 1, or a footnote to reiterate that percentages are not comparable row-wise.

Thank you for highlighting we have made the following changes in response to your comment, to add clarity. Label for total population row has had (denominator population) added to make explicit that the denominator populations are different and a footnote has been added to aid interpretation.

"Nb. Due to different population denominators percentages are within columns but not row-wise."

9. Model results: It would be helpful to re-iterate the statistical model applied before getting into the results, e.g. a single sentence reminding readers of what was modelled on what, and to confirm the N (I assume 1.2 million if imputation was used, but it would be clearer to confirm directly in-text and/or in the relevant tables.

Sentence added to results section for clarity:

"A multi-level regression model was used with missing data imputed using chained equations, so the entire population sample was used for analysis"

10. Table 3: There is an incomplete parenthesis in the footnote.

Typo Has been corrected

11. Sensitivity analyses: This section seems weakly supported as no tables of results are provided. Additionally, it is unclear if any of the reported findings relate to sensitivity analysis 4 (mortality restricted to 2019 to avoid Covid-19). It would significantly strengthen this section if supplementary tables could be provided.

Thank you for highlighting this. The results from the sensitivity analyses have been added in supplementary tables S3, S4, S5, and S6.

The mortality analysis that was restricted to 2019 was to examine whether Covid mortality had had an impact on the results.

Discussion:
12. The discussion is supported by the data, the key caveats are noted and the conclusions valid. However, the study has extremely high statistical power which means that significance testing is less informative, and I notice that the effect size or clinical significance of the effect estimates is not commented on. Adding some comment on effect magnitude could add more detail to the interpretation.

A couple of sentences added, noting that that clinical significance for the individual patient is small, however impact on the primary care system as a whole is significant.

"There are 17.4 million middle aged adults in the UK. With 13.9% of these adults experiencing PIP and there being an associated 3% increase in GP consultations, this amounts to an extra 362,790 consultations a year (~one in every thousand GP appointments being attributable to PIP in middle aged adults). Although at an individual level this increased utilisation may not impact the patient, it is a significant increase in demand on a stretched primary healthcare system."

VERSION 2 - REVIEW

**Reviewer**	**1**
**Name**	**mitchell, aaron**
**Affiliation**	**Memorial Sloan Kettering Cancer Center**
**Date**	**18-Jun-2026**
**COI**	

I have only one, minor comment regarding this revised manuscript. I had previously made a comment about the ethnicity categorizations which I believe may have been misinterpreted. I did not mean to raise any concerns about how the groups were constructed, or about the heterogeneity within them. It was solely a question of terminology. In the United States, groupings such as “white” and “black” would be referred to as race categories, rather than ethnicity categories. With the rationale being that these labels often have little to do with a person’s ancestry or country of origin, and are applied based on socially constructed ideas of what a person “looks like.” Since this is solely a language/terminology question rather than a scientific one, I would fully defer to the journal editorship on what the proper term would be, especially if the standards for these terms are different in the UK vs. US.

All of my other questions have been adequately addressed in this revision.
